# Two-Channel VO_2_ Memory Meta-Device for Terahertz Waves

**DOI:** 10.3390/nano11123409

**Published:** 2021-12-16

**Authors:** Xueguang Lu, Bowen Dong, Hongfu Zhu, Qiwu Shi, Lu Tang, Yidan Su, Cheng Zhang, Wanxia Huang, Qiang Cheng

**Affiliations:** 1 College of Materials Science and Engineering, Sichuan University, Chengdu 610065, China; luxueguang@stu.scu.edu.cn (X.L.); zhulangren1110@163.com (H.Z.); shiqiwu@scu.edu.cn (Q.S.); scjdlnmkj@163.com (L.T.); 2 Department of Basic Sciences, Air Force Engineering University, Xi’an 710051, China; dongdeduo@163.com; 3 School of Engineering, The University of Manchester, Manchester M13 9PL, UK; ethan_su@126.com; 4 Hubei Engineering Research Center of RF-Microwave Technology and Application, School of Science, Wuhan University of Technology, Wuhan 430070, China; 5 State Key Laboratory of Millimeter Waves, Department of Radio Engineering, Southeast University, Nanjing 210096, China

**Keywords:** meta-memory, terahertz, vanadium dioxide, reconfigurable metasurface

## Abstract

Vanadium oxide (VO_2_), as one of the classical strongly correlated oxides with a reversible and sharp insulator-metal transition (IMT), enables many applications in dynamic terahertz (THz) wave control. Recently, due to the inherent phase transition hysteresis feature, VO_2_ has shown favorable application prospects in memory-related devices once combined with metamaterials or metasurfaces. However, to date, VO_2_-based memory meta-devices are usually in a single-channel read/write mode, which limits their storage capacity and speed. In this paper, we propose a reconfigurable meta-memory based on VO_2_, which favors a two-channel read/write mode. Our design consists of a pair of large and small split-ring resonators, and the corresponding VO_2_ patterns are embedded in the gap locations. By controlling the external power supply, the two operation bands can be controlled independently to achieve at least four amplitude states, including “00”, “01”, “10”, and “11”, which results in a two-channel storage function. In addition, our research may provide prospective applications in fields such as THz switching, photon storage, and THz communication systems in the future.

## 1. Introduction

Terahertz (THz) waves refer to electromagnetic waves with a frequency of 0.1–10 THz that are located between millimeter waves and the infrared region. THz waves show enormous potential for application in the fields of high-speed wireless communication [1], imaging [2], and non-destructive testing [3] due to their merits, such as broadband, transient (picosecond magnitude), and low photon energy.

To promote the development of the abovementioned applications, it is necessary to achieve fast and efficient dynamic modulation of THz waves. Therefore, dynamic control of THz waves has become an in-depth research topic. In particular, metamaterials or metasurfaces that integrate with some functional materials have led to significant achievements in THz dynamic manipulations [4,5,6]. When excited by external physical fields, functional materials such as doped semiconductor [7,8,9], graphene [10,11,12], ferroelectric [13,14], MXene [15,16], phase change materials [17,18,19], and the emerging piezoelectrically-addressable polymer [20,21] can directly affect the response characteristics of the hybrid meta-structures to THz waves. Among these smart materials, the phase change material vanadium oxide (VO_2_) has frequently attracted much attention due to its outstanding features. It is worth noting that the insulator-metal transition (IMT) of VO_2_ can be modulated arbitrarily by multiphysical external fields such as heating [22,23], applied currents [24,25], laser pumping [26], and even straining [27]. Additionally, its dynamic state can be maintained after the external stimulus returns to the critical excitation point as a result of the inherent metastability of the first-order phase transition (the so-called “memory effect”). On the basis of these two merits, various memory-type devices have been proposed by making use of pure VO_2_ films or VO_2_-based meta-structures. For example, Driscoll et al. [28] first demonstrated a memory response in VO_2_ thin films through electronic control. Subsequently, similar memory models based on pure VO_2_ film were successively revealed, and the corresponding experiments termed “electric writing” [29] and “optical writing” [30] were carried out as predicted [29,30,31,32]. However, pure VO_2_ films are not easy to design for practical applications, and the storage capacity of the device is limited by the condition that the signal state must be read clearly. In the past decade, metamaterials or metasurfaces have emerged as an effective means of engineering THz waves, paving a new way to realize memory-related devices with high performance. Different from the abovementioned memorizers made by pure VO_2_ film, the coupling of metamaterial or metasurface with VO_2_ patterns can help enhance the contrast between different memorial states, as well as make a significant simplification of the memory operations. For instance, an electrically controlled, tunable metasurface based on the VO_2_ memory effect was proposed in [33], which can achieve polymorphic memory storage in the THz range. However, the whole system used external heating as auxiliary means to control the phase change process, so the reading and writing speed was slow. Furthermore, Cai et al. implemented a multifunctional memory storage meta-device in the THz band to realize writing and erasing functionalities between multiple states through current control instead of relying on an external heat source [34]. A similar strategy has also been adopted within the infrared light region [35].

Despite the rapid development of memory-related applications, there is still a shortcoming of the existing meta-devices in memory density because of the single-channel read/write mode. To address this problem, in this paper, we propose a reconfigurable metasurface, which integrates VO_2_ patterns with a pair of large and small metallic split ring resonators (SRRs), which enables dynamic storage of information independently in two channels. Note that each column of SRRs shares one feed line to control the input current; therefore, the coded current can independently adjust the transmittance at 0.32 and 0.362 THz. The maximum modulation depth of our design can reach 89.3% (0.32 THz) and 85.0% (0.362 THz) in the simulation, respectively. Hence, if the high (termed state “1”) and low (termed state “0”) amplitude states are taken as the stored information, at least four storage states of “00”, “01”, “10”, and “11” can be combined in the two frequency bands. Compared to the previous single-channel design, the two-channel independent read/write mode allows for more flexible and diverse storage forms and storage states. This research can be used to inspire the design of tunable multifunctional devices in the THz range.

## 2. Theory

**Figure 1:**
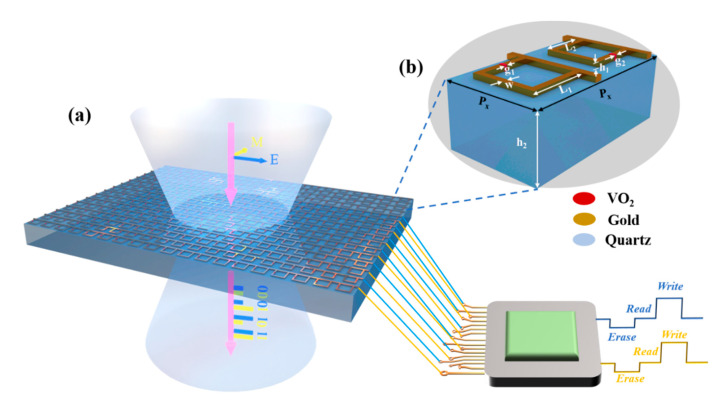
Schematic diagram of the two-channel THz memory storage device based on vanadium dioxide. (**a**) Schematic diagram of a reconfigurable metasurface in which two electrodes input a programmable current to independently control the THz transmission state of two frequency channels. (**b**) Unit cell of the metasurface is depicted in the inset, where *P*_x_ = 220 μm, *P*_y_ = 120 μm, *L*_1_ = 80 μm, *L*_2_ = 70 μm, *g*_1_ = 4 μm, *g*_2_ = 4 μm, *h*_1_ = 0.2 μm, and *h*_2_ = 200 μm.

Figure 1a shows a schematic diagram of a two-channel THz storage device based on VO_2_, achieving independent transmission control at 0.32 THz and 0.362 THz by encoding an external feed current. Moreover, the storage function is realized based on the intrinsic phase change memory effect of VO_2_. As depicted in Figure 1b, the meta-atom comprises two layers: the upper layer, which has large and small metal opening rings and VO_2_ patterns embedded in the gaps, and the bottom layer, which is a quartz substrate. The periodicity of our subwavelength meta-atoms is *P*_x_ = 220 μm and *P*_y_ = 120 μm. The other geometrical parameters are *L*_1_ = 80 μm, *L*_2_ = 70 μm, *g*_1_ = *g*_2_ = 4 μm, *w* = 9 μm, *h*_1_ = 0.2 μm, and *h*_2_ = 200 μm. The thickness of the VO_2_ is set to 0.17 μm according to the actual preparation situation.

At room temperature, the dielectric constant of VO_2_ is approximately 9 in the insulating state, and by applying an external bias current, structural transformation occurs, turning VO_2_ into the metallic phase. Because in polycrystalline VO_2_ films, the phase transition starts at a seed point in the film, and as the temperature increases, metal domains grow and diffuse around or above the phase transition temperature. As a result, a coexistence of semiconductor and metal domains can occur. The spatial inhomogeneity of the film influences the effective dielectric properties of the film [36,37], and the complex dielectric properties *ε*_eff_ of VO_2_ can be characterized by the Bruggeman effective-medium theory in the simulation [36,37,38,39,40,41,42].

(1) $$f_{m}\frac{\varepsilon_{m}-\varepsilon_{\mathrm{eff}}}{\varepsilon_{m}+(d-1)\varepsilon_{\mathrm{eff}}}+f_{i}\frac{\varepsilon_{i}-\varepsilon_{\mathrm{eff}}}{\varepsilon_{i}+(d-1)\varepsilon_{\mathrm{eff}}}=0$$

where *ε*_i_ and *ε*_m_ denote the dielectric constants of the semiconductor and metallic regions, respectively. In addition, *f*_m_ and *f*_i_(*f*_m_ + *f*_i_ = 1) represent volume fractions of the metallic and insulating grains, respectively. The dimensionality of the composite medium *d* was set to 2 [40]. To evaluate the complex dielectric properties of the mixture, Equation (1) must be expressed as a function of *ε*_i_, *ε*_m_, and *f*_m_. Rearranging (1) and solving for the quadratic results in:

(2) $$\varepsilon_{\mathrm{eff}}=\frac{1}{4}\left\{\varepsilon_{i}(2-3f_{m}+\varepsilon_{m}(3f_{m}-1)+\sqrt{{\left[\varepsilon_{i}(2-3f_{m})+\varepsilon_{m}(3f_{m}-1)\right]}^{2}+8\varepsilon_{i}\varepsilon_{m}}\right\}$$

where the volume fraction *f*_m_ of the metal region can be described as [43]:

(3) $$f_{m}=1-\left\{1+\exp((T-T_{0})/\Delta T)\right\}$$

where *T*_0_ and ∆*T* are the phase transition temperature and the hysteresis temperature width of the MIT. Therefore, by combining Equations (2) and (3), we can determine the equivalent dielectric constant of the VO_2_ film at different temperatures. In addition, quartz can be considered a lossy dielectric, with a dielectric constant of 3.75 + 0.0004i, and the gold structure is taken as a lossy metal with a conductivity of 4.56 × 10^7^ S/m.

## 3. Materials and Devices

**Figure 2:**
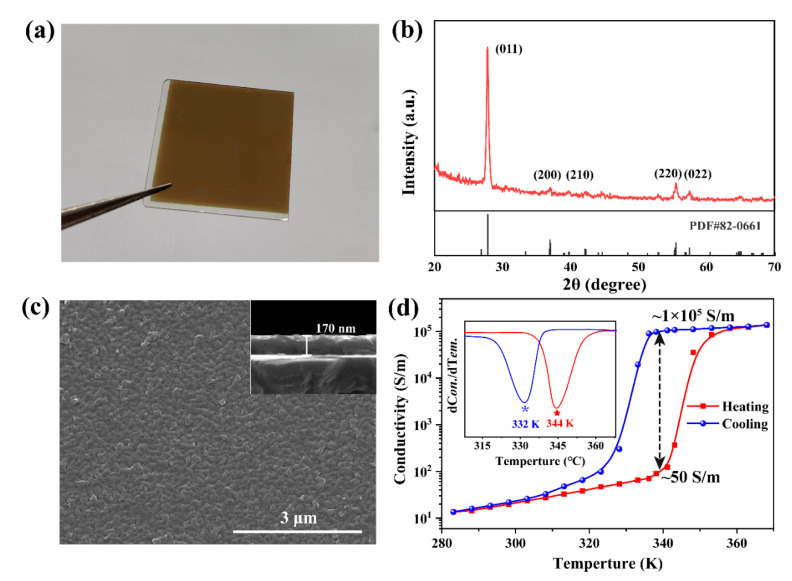
Preparation and characterization of VO_2_ films. (**a**) Optical image of the VO_2_ thin film prepared by magnetron sputtering. (**b**) XRD pattern and (**c**) SEM image of the VO_2_ film. (**d**) Conductivity-temperature curves for VO_2_ films grown on SiO_2_ substrate.

In this work, VO_2_ film samples were prepared by reactive radio frequency magnetron sputtering deposition. A metal vanadium target (purity of 99.9%, diameter of 2 inches, thickness of 5 mm, Zhongnuo New Materials (Beijing) Technology Co., Ltd., Beijing, China) was used to deposit VO_2_ film on silicon dioxide (SiO_2_) substrate. In the experiments, the distance between the target and the substrate was about 15 cm. The working gas, Ar with 99.999% purity, and the reaction gas, O_2_ with 99.999% purity, were introduced into the chamber with two mass flow controllers under a background vacuum of 8 × 10^−4^ Pa. The target was pre-sputtered for 5 min to eliminate the target surface contamination. During the deposition process, the Ar and O_2_ flow ratio was set to 60/2.4 sccm, and the total partial pressure of the gas in the chamber was ~0.7 Pa. The deposition temperature was maintained at 600 °C, the sputtering time was 40 min, and the sputtering power was 140 W. The optical photograph of the prepared film is shown in Figure 2a, which is a macroscopically transparent yellow-brown color.

The X-ray diffraction (XRD, D/MAX2500, Tokyo, Japan) patterns of the films were collected with Cu Kα as the source of radiation (*λ* = 0.15406 nm) under grazing incidence at an angle of 1.5°. As shown in Figure 2b, the peaks at approximately 27.98°, 37.12°, 42.34°, 55.64°, and 57.64° are indexed to diffractions from the (011), (200), (210), (220), and (022) planes of the monoclinic VO_2_ phase, respectively (JCPDS card no. 82-0661). The XRD pattern indicates good crystallinity with a preferred orientation for the film, and the relatively strong peak at approximately 27.98° indicates a preferred orientation of (011) for the films. The surface morphology of the film was observed with a field-emission scanning electron microscope (FEI Inspect F50, New York, NY, USA). The scanning electron microscopy (SEM) image of the VO_2_ film shown in Figure 2c illustrated that the VO_2_ film is compact without noticeable pores and consists of uniform, continuous nanoparticles whose size ranges from 50 to 90 nm with a mean value of approximately 70 nm. The inset at the upper right shows that the thickness of the prepared film is approximately 170 nm.

The electrical phase transition characteristics of VO_2_ thin films were studied by using the four-point probe method. The change in film sheet resistance with temperature was recorded, and the change in sample conductivity was calculated by combining the thickness of the film (Figure 2d). It can be seen from the figure that the conductivity value of the sample does not coincide in the process of heating and cooling, thus forming a hysteretic curve, which is one of the characteristics of the VO_2_ phase transition. When the temperature of the sample rose from 298 K to 363 K, the conductivity of the sample changed from 23 S/m to 1.2 × 10^5^ S/m. The maximum hysteresis interval exists at approximately 341 K. The heating and cooling curves correspond to conductivities of ~50 S/m and ~1 × 10^5^ S/m, respectively, spanning nearly 4 orders of magnitude. This change also indicates that our film prepared on SiO_2_ has a very high quality.

## 4. Results and Discussion

**Figure 3:**
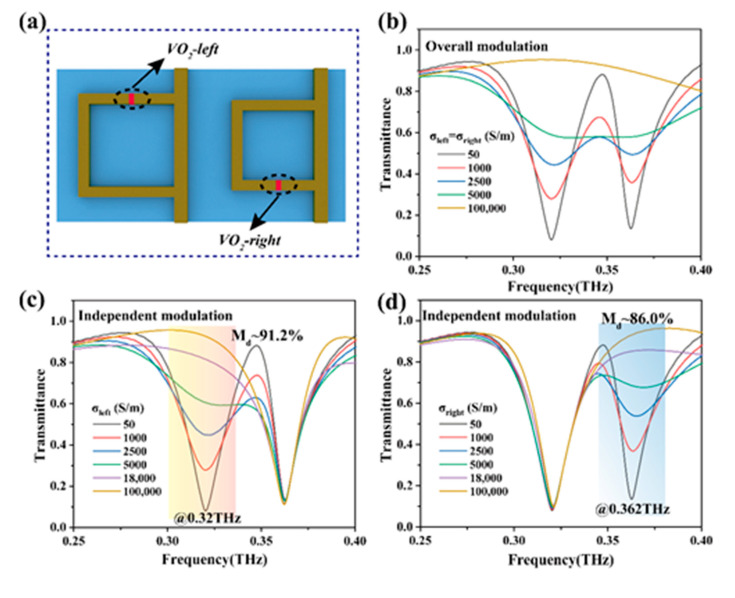
Effect of conductivity variation on terahertz transmittance at resonant peak at two frequency bands during VO_2_ phase transition. (**a**) In the top view of the unit structure, two VO_2_ pieces are independently embedded in the large and small opening rings. (**b**) When the conductance of the VO_2_ film on the left and right sides of the unit changes simultaneously, the terahertz transmittance at the resonant peak of 0.25 THz and 0.362 THz increases synchronously. (**c**,**d**) Keep the VO_2_ conductivity on one side constant and the VO_2_ on the other side gradually changes. (**c**) Right side and (**d**) left side VO_2_ conductivity unchanged.

In this work, we simulated the transmission spectra of the hybrid metasurface under *x*-polarized incidence using the commercial software CST Microwave Studio. To study the influence of VO_2_ conductivity on the two designed terahertz channels, the changes in the transmittance at 0.32 THz and 0.362 THz in the target frequency band during the change of VO_2_ conductivity from 50 S/m to 1 × 10^5^ S/m were calculated, respectively. As shown in Figure 3a, VO_2_ patterns are distributed in the gaps of the left and right opening rings of different sizes, and the magnitude of the input current controls the degree of phase transformation. When VO_2_ patterns were in the insulating state (50 S/m), in the absence of external stimulation, the metasurface array had a resonance response at 0.32 THz and 0.362 THz, respectively. When the phase transformation degree of VO_2_ patterns was controlled to increase at the left and right sides, the transmittance at 0.32 THz increased from 8.1% to 92.6%, and at 0.362 THz, the transmittance increased from 13.0% to 90.0% (Figure 3b). Therefore, a high THz wave modulation depth can be obtained at two resonant peaks during the VO_2_ phase transition process.

**Figure 4:**
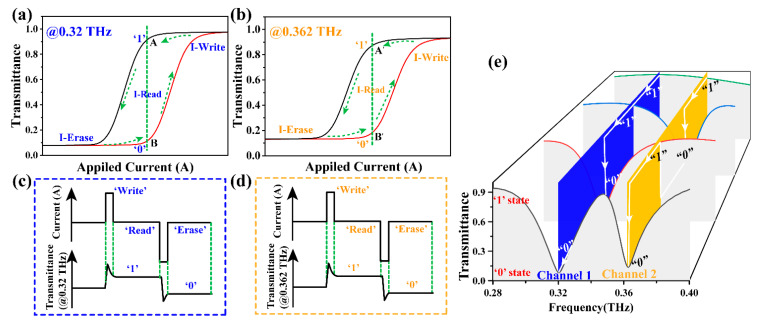
Effect of conductivity variation on terahertz transmittance at resonant peak at two frequency bands during VO_2_ phase transition. (**a**) In the top view of the unit structure, two VO_2_ pieces are independently embedded in the large and small opening rings. (**b**) When the conductance of the VO_2_ film on the left and right sides of the unit changes simultaneously, the terahertz transmittance at the resonant peak of 0.25 THz and 0.362 THz increases synchronously. (**c**,**d**) Keep the VO_2_ conductivity on one side constant and the VO_2_ on the other side gradually changes. (**c**) Left side and (**d**) right side VO_2_ conductivity unchanged. (**e**) Schematic diagram of the two channels being stored independently.

In addition, the transmittance of the two resonant peaks can be adjusted independently, and the phase transformation of VO_2_ patterns in the gaps of the large or small opening rings can be controlled by feeding separately. As shown in Figure 3c,d, when VO_2_ (located on the left) in the gap of the large opening ring was stimulated by external current while VO_2_ at the small opening ring remained insulated, the obtained spectral transmittance at 0.32 THz increased gradually (from 8.1% to 92.6%), while the transmittance at 0.362 THz remained unchanged. Instead, the VO_2_ in the array with the small opening ring (on the right side of the whole) was excited, and the VO_2_ at the large opening ring remained insulated. The result was that the transmittance at 0.362 THz was modulated (from 13% to 92.8%), while the transmittance at 0.32 THz remained the same. The amplitude modulation depth (*M_d_*) is defined as $M_{d}=\left(T_{\max}-T_{\min}\right)/T_{\max}$. Therefore, approximately 91.2% and 86.0% of the modulation depth can be obtained at the 0.32 and 0.362 THz channels, respectively. VO_2_ has phase transformation hysteresis under external stimuli, such as thermal, electrical, photothermal and other conditions (Figure 2d), which can be used in memory devices. The cyclic hysteresis data of the conductance phase change of the VO_2_ thin film in Figure 2d were extracted and imported into CST for simulation calculation to obtain the corresponding transmission hysteresis curves at 0.32 THz and 0.362 THz, as shown in Figure 4a,b. Due to the optical hysteresis behaviors demonstrated, the meta-device can realize electric-controlled memory by utilizing the intrinsic hysteretic behavior of VO_2_. For example, looking at the transmission change of 0.32 THz in Figure 4a, the current at the maximum hysteretic point is selected as the “Read” current input. At that moment, a short current pulse is applied based on the “Read” current as the “Write” current, which can quickly cause the phase transition of VO_2_ and increase the transmission (as shown in the “Write” current pulse in Figure 4c). When returning to the “Read” current, the high transmission state can still be maintained (point A), that is, the “1” state at 0.32 THz. Here, for convenience, the high transmission state of terahertz is the “1” state, and the relatively low transmission state is the “0” state. If the input of the “Read” current is reduced rapidly over a brief period of time (as shown in the “Erase” current pulse in Figure 4c) then VO_2_ quickly recovers and results in a decrease in transmittance at 0.32 THz. When returning to the “Read” current, the low transmission state can be maintained (point B), that is, the “0” state at 0.32 THz. Therefore, the “0” and “1” states of 0.32 THz can be read and erased by encoding the current pulse, as shown in Figure 4b. Similarly, the resonance peak transmission at 0.362 THz can also be independently applied to the current for “0” and “1” state coding. Because the 0.32 THz and 0.362 THz bands can be modulated independently without interference, the two channels can be operated independently or combined to produce at least four different storage states of “00”, “01”, “10”, and “11”, which can significantly improve storage efficiency and flexibility. The four states here result from the cross-combination of the two channels. For example, “01” indicates a low transmission state at 0.32 THz and a high transmission state at 0.362 THz (Figure 4e). It is worth mentioning that the two-channel mode can be designed for 16 or more operating states when the contrast ratio is satisfactory (more details can be seen in Figure S1 of the Supporting Information). Currently, VO_2_-based terahertz storage devices are generally available in a single-channel mode. Therefore, based on this design, storage efficiency and flexibility can be significantly improved.

**Figure 5:**
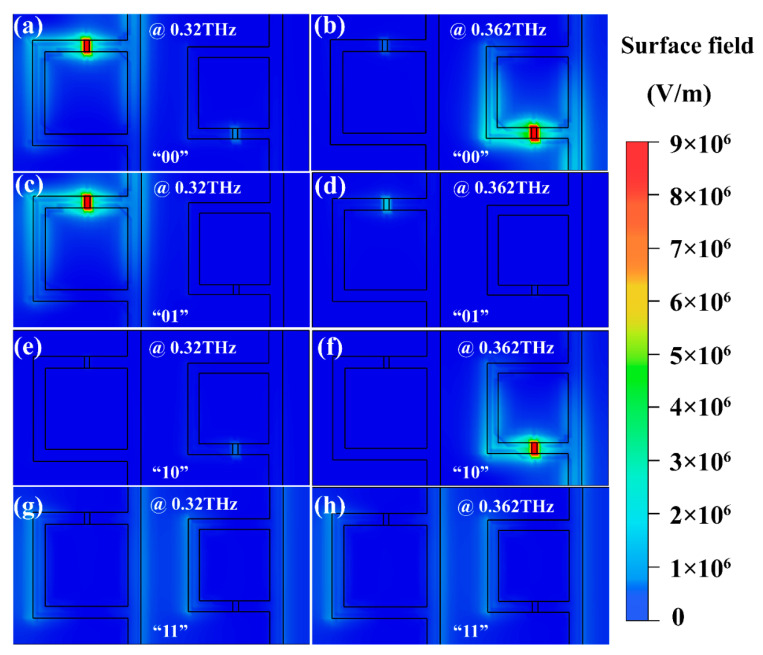
At 0.32 THz and 0.362 THz, respectively, the electric field distribution diagram of the cell structure in different storage states. (**a**,**b**) *σ*_left_ = *σ*_right_ = 50 S/m (“00” state); (**c**,**d**) *σ*_left_ = 50 S/m, *σ*_right_ = 100,000 S/m (“01” state); (**e**,**f**) *σ*_left_ = 100,000 S/m, *σ*_right_ = 50 S/m (“10” state); (**g**,**h**) *σ*_left_ = *σ*_right_ = 100,000 S/m (“11” state).

**Figure 6:**
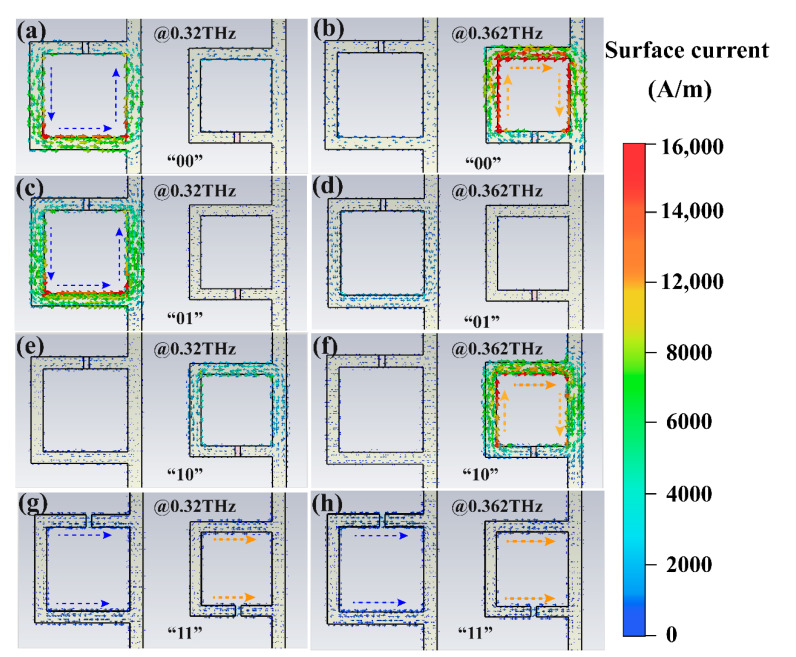
The 0.32 THz and 0.362 THz values, respectively, the surface current distribution diagram of the cell structure in different storage states. (**a**,**b**) *σ*_left_ = *σ*_right_ = 50 S/m (“00” state); (**c**,**d**) *σ*_left_ = 50 S/m, *σ*_right_ = 100,000 S/m (“01” state); (**e**,**f**) *σ*_left_ = 100,000 S/m, *σ*_right_ = 50 S/m (“10” state); (**g**,**h**) *σ*_left_ = *σ*_right_ = 100,000 S/m (“11” state).

To further understand the mechanism of the influence of VO_2_ conductivity change on resonance, the electric field and current distribution on the structure surface under different storage states (“00”, “01”, “10”, and “11”) are shown in Figure 5 and Figure 6, respectively. At 0.32 THz, when VO_2_ patterns in the gaps between the large and small rings were insulating, the electric field concentrated on the opening of the large ring, and a strong ring current was formed on the surface (Figure 5a and Figure 6a). At this moment, the resistance of the VO_2_ patterns was large and equivalent to that of a capacitor, which is a typical L-C resonant mode. Consequently, the transmittance at 0.32 THz was low. Similarly, a ring current was also formed on the surface of the small ring, but the structure size was small, and the resonance caused by it appeared at 0.362 THz (Figure 5b and Figure 6b). While the VO_2_ patterns embedded in the large ring openings remained insulated, the conductivity of the VO_2_ patterns in the small ring openings began to rise in response to environmental stimuli. The electric field intensity at the opening of the small rings decreased and the surface ring current decreased, resulting in an increase in the transmission intensity at 0.362 THz. However, at 0.32 THz, there was still a strong ring current on the surface of the large rings, indicating that there was still a strong L-C resonance, and so 0.32 THz was in the low transmission state. That is, the device was now in the “01” state.

In contrast, Figure 5e,f and Figure 6e,f show the electric field distribution in the “10” state. In this case, there was a substantial ring current on the surface of the small rings at 0.362 THz and a concentration of electric field at the openings, so the transmission at 0.362 THz was low. While the VO_2_ conductivity at the large rings increased, the capacitance property at the opening weakened, and the transmittance at 0.32 THz increased. When the VO_2_ patterns in the large and small opening rings underwent phase transformation, there was no electric field concentration on the surface of the two structures, and the surface current was relatively weak, as shown in Figure 5g,h and Figure 6g,h. This situation corresponds to the “11” state.

## 5. Conclusions

In this work, we have demonstrated a tunable memory meta-device based on the IMT of VO_2_, in which large and small splitting rings are arranged alternatively, and VO_2_ patterns are embedded in the opening part of the splitting ring. Under the applied coding current, the transmittance of 0.32 THz and 0.362 THz can be controlled independently, and modulation depths of 91.2% and 86.0% can be obtained, respectively. Most importantly, based on the intrinsic phase change memory effect of VO_2_, at least four storage states of “00”, “01”, “10” and “11” can be attained by encoding the high and low terahertz transmission states at the 0.32 and 0.362 THz channels. In addition, based on this design, the storage capacity can be increased exponentially with the number of coded channels. Compared with the previous single-channel storage mode, the design can significantly improve storage efficiency and flexibility. Due to these characteristics, the proposed meta-device may provide a reference for the design of tunable devices, terahertz switches and photonic storage devices in the terahertz range.

## Data Availability

The data presented in this study are available on request from the corresponding author.

## Supplementary Materials

The following are available online at https://www.mdpi.com/article/10.3390/nano11123409/s1, Figure S1: Demonstration of 16 storage status functions in two-channel mode.
