# Supplementary material for: Two-Channel VO2 Memory Meta-Device for Terahertz Waves
Source: Nanomaterials (Basel). 2021 Dec 16;11(12):3409. doi: 10.3390/nano11123409 (PMC8705468; doi:10.3390/nano11123409)
Supplement: Supplementary file 1 [file nanomaterials-11-03409-s001.zip › nanomaterials-1483221-supplementary.pdf]

nanomaterials

MDPI

Supplementary Material

Two-Channel VO_2 Memory Meta-Device for Terahertz Waves

Xueguang Lu [1], Bowen Dong [2], Hongfu Zhu [1], Qiwu Shi [1], Lu Tang [1], Yidan Su [3], Cheng Zhang [4,*], Wanxia Huang [1,*] and Qiang Cheng [5,*]

1 College of Materials Science and Engineering, Sichuan University, Chengdu 610065, China; luxueguang@stu.scu.edu.cn (X.L.); zhulangren1110@163.com (H.Z.); shiqiwu@scu.edu.cn (Q.S.); scjdlnmkj@163.com (L.T.)
2 Department of Basic Sciences, Air Force Engineering University, Xian 710051, China; dongdeduo@163.com
3 School of Engineering, The University of Manchester, Manchester M13 9PL, UK; ethan_su@126.com
4 Hubei Engineering Research Center of RF-Microwave Technology and Application, School of Science, Wuhan University of Technology, Wuhan 430070, China
5 Department of Radio Engineering, State Key Laboratory of Millimeter Waves, Southeast University, Nanjing 210096, China
* Correspondence: czhang2020@whut.edu.cn (C.Z.); huangwanxia@scu.edu.cn (W.H.); qiangcheng@seu.edu.cn (Q.C.); Tel.: +86-028-8540-5781 (W.H.)

Section 1 Demonstration of 16 storage status functions in two-channel mode

The polymorphic memory effect of VO_2 has been reported before [1,2]. As shown in Figure S1, the conductivity change paths of VO_2 films after cooling to 368 K, 346 K, 343 K, and 242 K are different, which is helpful for multistate storage. Moreover, the two-channel design can further exploit this advantage. The conductivity changes of the heating curve and several cooling curves (368 K, 343 K and 342 K) in Figure S1a are extracted and simulated in CST to obtain the transmittance hysteresis curves at 0.32 THz and 0.362 THz, respectively. Therefore, four transmittance states can be obtained in each frequency band by controlling the "Write" current, i.e., "00", "01", "10" and "11". Since the modulations at 0.32 and 0.362 THz are independent of each other, a total of 16 storage states can be obtained by combining the transmission states of the two bands, as shown in Figure S1d. Compared with the single-channel design, the two-channel mode has improved flexibility and storage density.

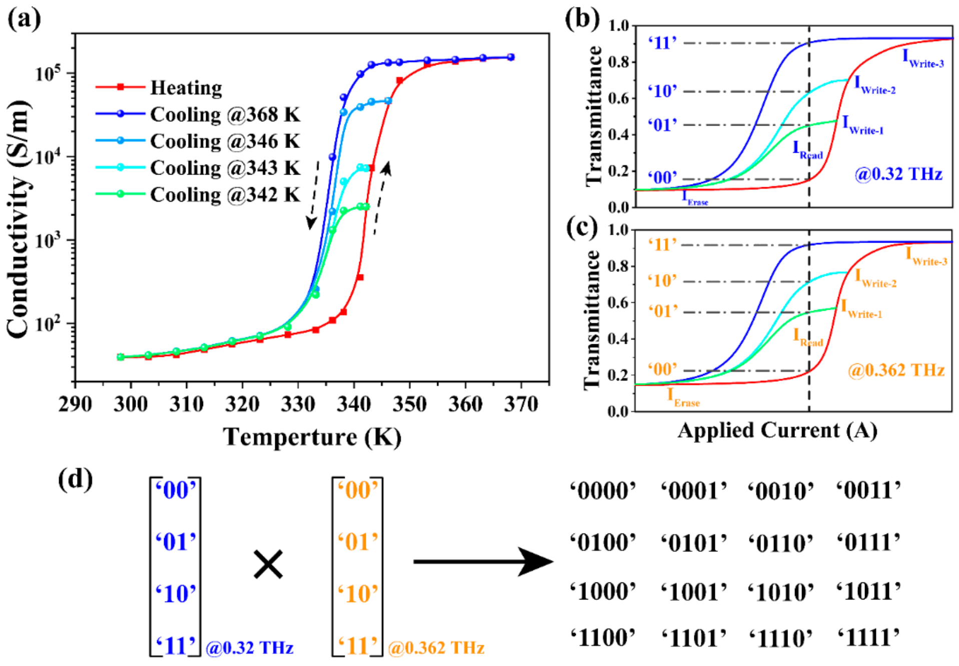

Figure S1. (**a**) A series of hysteretic curves of the conductivity-temperature of the VO_2 film show that the temperature drops from 368 K, 346 K, 343 K and 342 K. (**b-c**) The variation in conductance during the phase transition of VO_2 was extracted to simulate the hysteretic characteristics of the transmission amplitude at 0.32 THz and 0.362 THz with the cyclic change of the applied current. (**d**) Sixteen storage states were obtained by cross-combining the amplitude information at 0.32 and 0.362 THz.

2. Mechanism of electrogenic insulator-metal transition (IMT) in VO_2 thin films

The phase transition hysteresis characteristics of VO_2 are indeed very interesting phenomena [3], but the main mechanism of the voltage-/current-driven insulator-metal transition (IMT) process in VO_2 films is still under dynamic study and remains controversial. For example, Zimmers *et al.* [4] claimed that local Joule heating plays a predominant role in DC voltage/current-induced IMT in VO_2 thin wires. In contrast, Wu *et al.* [5] believed that the Joule heating effect was insignificant and that the electric field alone was sufficient to induce IMT. Beyond these two competing claims, some articles have reported that it is difficult to disentangle these two effects in the voltage/current driven IMT process in VO_2 thin wires [6,7]. In spite of the controversy on mechanism, it does not prevent the application of VO_2 in device research. A large number of experiments have confirmed its feasibility in the field of electrically controlled terahertz wave dynamic modulation.

References

1. Driscoll, T.; Kim, H.-T.; Chae, B.-G.; Di Ventra, M.; Basov, D.N. Phase-Transition Driven Memristive System. *Appl. Phys. Lett.* **2009**, *95*, 043503.
2. Coy, H.; Cabrera, R.; Sepúlveda, N.; Fernández, F.E. Optoelectronic and All-Optical Multiple Memory States in Vanadium Dioxide. *Journal of Applied Physics.* **2010**, *108*, 113115.
3. del Valle, J.; Salev, P.; Tesler, F.; Vargas, N.M.; Kalcheim, Y.; Wang, P.; Trastoy, J.; Lee, M.-H.; Kassabian, G.; Ramírez, J.G.; et al. Subthreshold Firing in Mott Nanodevices. *Nature* **2019**, *569*, 388–392.
4. Zimmers, A.; Aigouy, L.; Mortier, M.; Sharoni, A.; Wang, S.; West, K.G.; Ramirez, J.G.; Schuller, I.K. Role of Thermal Heating on the Voltage Induced Insulator-Metal Transition in VO_2. *Phys. Rev. Lett.* **2013**, *110*, 056601.
5. Wu, B.; Zimmers, A.; Aubin, H.; Ghosh, R.; Liu, Y.; Lopez, R. Electric-Field-Driven Phase Transition in Vanadium Dioxide. *Phys. Rev. B.* **2011**, *84*, 241410.
6. Joushaghani, A.; Jeong, J.; Paradis, S.; Alain, D.; Stewart Aitchison, J.; Poon, J.K.S. Voltage-Controlled Switching and Thermal Effects in VO_2 Nano-Gap Junctions. *Appl. Phys. Lett.* **2014**, *104*, 221904.
7. Liao, G.M.; Chen, S.; Fan, L.L.; Chen, Y.L.; Wang, X.Q.; Ren, H.; Zhang, Z.M.; Zou, C.W. Dynamically Tracking the Joule Heating Effect on the Voltage Induced Metal-Insulator Transition in VO_2 Crystal Film. *AIP Advances.* **2016**, *6*, 045014.
